# Advanced Insights into Functional Brain Connectivity by Combining Tensor Decomposition and Partial Directed Coherence

**DOI:** 10.1371/journal.pone.0129293

**Published:** 2015-06-05

**Authors:** Britta Pester, Carolin Ligges, Lutz Leistritz, Herbert Witte, Karin Schiecke

**Affiliations:** 1 Bernstein Group for Computational Neuroscience Jena, Institute of Medical Statistics, Computer Sciences and Documentation, Jena University Hospital, Friedrich Schiller University Jena, Bachstraße 18, Jena, Germany; 2 Department of Child and Adolescent Psychiatry, Psychosomatic Medicine and Psychotherapy Jena University Hospital, Friedrich Schiller University Jena, Philosophenweg 3–5, Jena, Germany; Wadsworth Center, UNITED STATES

## Abstract

Quantification of functional connectivity in physiological networks is frequently performed by means of time-variant partial directed coherence (tvPDC), based on time-variant multivariate autoregressive models. The principle advantage of tvPDC lies in the combination of directionality, time variance and frequency selectivity simultaneously, offering a more differentiated view into complex brain networks. Yet the advantages specific to tvPDC also cause a large number of results, leading to serious problems in interpretability. To counter this issue, we propose the decomposition of multi-dimensional tvPDC results into a sum of rank-1 outer products. This leads to a data condensation which enables an advanced interpretation of results. Furthermore it is thereby possible to uncover inherent interaction patterns of induced neuronal subsystems by limiting the decomposition to several relevant channels, while retaining the global influence determined by the preceding multivariate AR estimation and tvPDC calculation of the entire scalp. Finally a comparison between several subjects is considerably easier, as individual tvPDC results are summarized within a comprehensive model equipped with subject-specific loading coefficients. A proof-of-principle of the approach is provided by means of simulated data; EEG data of an experiment concerning visual evoked potentials are used to demonstrate the applicability to real data.

## Introduction

Quantification of directed information transfer in complex brain networks has been one of the most fundamental challenges within the field of neuroscience in the past few decades [1]. A popular and well-established measure of connectivity is provided by time-variant partial directed coherence (tvPDC), which is calculated based on the Fourier transform of time-variant multivariate autoregressive (tvMVAR) model parameters [2–4]. Oftentimes, the frequential and/or temporal variety of neuronal information transfer is of special interest. In such cases it is necessary to use tvPDC rather than measures without frequency and/or time selectivity.

A serious drawback of tvPDC however is the immense amount of analysis output. In formal terms, the output of tvPDC analysis for one single subject is a three-way data array (tensor) containing the modes space, time and frequency. More practically speaking, the information about directed information transfer between two nodes (e.g. EEG electrodes) is provided by two time-frequency maps of tvPDC values; the multivariate tvPDC output for a network of *D* nodes is consequently expanded to *D*(*D* − 1) time-frequency maps. If there are only a few nodes that exist in the network or that are of interest, the inspection of all tvPDC maps offers an overall view of the whole network. However, in clinical practice it is oftentimes impossible to predefine relevant nodes and the size of the complete network is usually too large to allow for a conjoint examination of the global network structures due to the quadratically (with *D*) increasing number of tvPDC maps that have to be inspected. To avoid this interpretational problem, the dimensionality of connectivity results is usually reduced by merging them over several time intervals and/or frequency ranges [5]. Indeed, this offers an opportunity to simplify the detection of general patterns, but can also diminish or even destroy the benefit of time variance and/or frequency selectivity.

In this work, we propose a linear decomposition of multi-way tvPDC tensors into their modes in order to assess a complementary view on connectivity results, facing the challenging handling of copious analysis output.

The concept of separating a multi-way tensor into a sum of rank-1 outer products is not new; first proposed in 1927 [6], it was adapted for psychometrics in 1970 [7, 8]. In the field of EEG it has been used to broaden traditional two-way decompositions such as Principal or Independent Component Analysis (PCA, ICA) to encompass multi-way data [9]. For EEG data, PCA and ICA provide a helpful means to extract event-related potentials from raw data [10–13]. By using these techniques however, analysis of several subjects is not possible without further effort: due to the problem of correspondence it is not clear if there is any component of subject A which corresponds to a certain one of subject B and even if there is one, detection of matching components is not trivial. By considering the data as a tensor with the modes space, time and subject, a factor decomposition results in the spatial and temporal loadings (as in PCA/ICA), together with an additional individual subject loading [14–16]. Similarly, this procedure can be used for comparison of different experimental conditions (e.g. task, stimulus, pre vs. post state) by adding the condition as an additional mode in the model [17].

In addition to the analysis of data in the time domain (data matrix of dimensionality channels × time), factorization can beneficially be used in the field of time-frequency analysis. When multivariate time-domain data are transferred into time-variant frequency space, as for example by Morlet wavelet transformation, they can be considered as a tensor with the third mode frequency. Thus, tensor decomposition can be used to linearly separate multivariate time-frequency information into the modes channels, time and frequency [18–20]. This enables a better identification, segregation and classification of frequency components which contribute to the signal. As delineated above for time-domain data, this procedure can analogously be extended to more than three modes if data of several subjects, groups, experimental conditions etc. are available.

We propose the application of tensor decomposition to tvPDC results in order to address the issue of deficient interpretability due to the vast number of results, supported by a segregation of underlying connectivity structures into different factors. This expands the decomposition of frequency transformed multivariate data to the decomposition of the degree of neuronal connectivity into its spatial, temporal and frequential content. Then, the spatial mode includes any ordered pair of channels where a connection is possible. Consequently, for *D* nodes, the spatial mode is of dimensionality *D* ∙ (*D* − 1). The factorization then drastically reduces the complexity of analysis outcome and thus provides a complementary perspective on the connectivity results. This enables an integrative view on tvPDC results in the first place. Furthermore, this reorganization of tvPDC results can contribute to uncovering inherent interaction structures that would possibly remain undetected if only raw tvPDC values were examined. Time-variant connectivity networks are usually composed of multiple components which emerge within different brain areas, time intervals and frequency bands. These separate underlying components can only be identified with difficulty by inspection of tvPDC, which can lead to basic interactions being missed. By approximating the tvPDC tensor as a sum of several factors, these components are separated and elementary patterns within the tvPDC results become more conspicuous.

Experimental data of the present proof-of-principle study were chosen in such way that they are associated with a strong working hypothesis. This offers the possibility to define a subset of electrodes which is of particular interest. Anyway, from a methodological point of view it is nevertheless necessary to perform a multivariate AR estimation of the whole set of electrodes in order to avoid spurious interactions arising from omitted nodes. Here, we propose the use of tensor decomposition for an extraction of connectivity patterns that are spatially limited to the channels of interest without disregarding the remaining electrodes. Therefore, as a first step, a full multivariate autoregressive model is estimated and tvPDC values are calculated. This retains mutual influences from any electrodes to the others. In a second step, subnetworks with a reduced number of nodes having exactly the same connections that appear in the full network over the reduced node set (so-called induced subnetworks [21]) are considered. The tensor decomposition is then applied to the tvPDC subnetwork tensors (based on full multivariate estimated AR parameters). This reorganization of tvPDC subsystems offers a supplementary view on the connectivity patterns within the considered subnetwork and can help to gain an insight into the subset-specific connectivity patterns in every mode.

Besides addressing the problematic issue of massive results, the tensor decomposition offers an opportunity to include a group of several subjects into the analysis. This challenging issue can be solved by adding the fourth mode subject, whereby every factor is additionally equipped with subject-individual weights.

To provide a proof-of-principle for our approach, we utilized simulated tvMVAR processes with a temporal, block-wise varying model structure. The applicability of tensor decomposition to real data is demonstrated on the basis of an exemplary EEG data set including 21 healthy subjects taken from a study investigating visual evoked potentials.

## Methods

### Time-variant AR and PDC computation

A *D*-dimensional time-variant multivariate autoregressive (tvMVAR) process with *N* sample points and order *p* is defined by

$$\text{Y}\left(n\right)=\overset{p}{\underset{r=1}{\sum }}A^{r}\left(n\right)\text{Y}\left(n-r\right)+\text{E}\left(n\right),n=p+1,\ldots ,N,$$

where $Y\left(n\right)\in R^{D}$ denotes the data vector of the *n*-th sample and the matrix $A^{r}(n)\in R^{D\times D}$ contains the *r*-th order AR parameters of sample *n*. The model residuals $E\in R^{D\times N}$ are supposed to be an uncorrelated *D*-dimensional Gaussian process with zero mean. In this work, time-variant MVAR estimation was performed by means of the multivariate linear Kalman Filter approach [22]. Basing on a multi-linear state space model for multi-trial time series, this algorithm integrates every trial separately and thus, data do not have to be averaged over trials before model estimation.

A frequency-selective, directed measurement of connectivity strength in tvMVAR models is provided by time-variant partial directed coherence (tvPDC). It is based on the Fourier transform of the AR process:

$$A\left(n,f\right)=I-\overset{p}{\underset{r=1}{\sum }}A^{r}\left(n\right){\text{e}}^{-2\pi ifr}\in {\mathbb{R}}^{D\times D},$$

with normalized frequency *f* ∈ [0,0.5] and identity matrix $I\in R^{D\times D}$. The degree of causal influence from node *j* to node *i* at sample *n* and frequency *f* can then be quantified by tvPDC, defined as

$$\pi_{i\leftarrow j}\left(n,f\right)≔\frac{\left|a_{ij}\left(n,f\right)\right|}{\sqrt{\sum_{d=1}^{D}{\left|a_{dj}\left(n,f\right)\right|}^{2}}}\in \left[0,1\right],i\neq j,$$

where *a*_*ij*_(*n*,*f*) denotes the (*i*,*j*)-th entry of **A**(*n*,*f*). Thus, for *F* considered frequency bins, the whole tvPDC tensor consists of (*D* ^2^ − *D*) ∙ (*N* − *p*) ∙ *F* entries.

### Tensor Factor Decomposition

In the following the theoretical background of parallel factor analysis (PARAFAC), which is the tensor decomposition approach proposed in [7] and [8] will be described. A detailed introduction can be found in [23, 24]. To lessen confusion regarding denotation, we mainly follow the nomenclature found in [20] and [23].

An *L*-th order tensor $X\in R^{I_{1}\times I_{2}\times \ldots \times I_{L}}$ is an ordered set of data $x_{i_{1},i_{2},\ldots ,i_{L}}$ with *L* indices. *L* is often also referred to as number of *modes* or *ways*. A one-way tensor $X\in R^{I}$ is a vector, a two-way tensor $X\in R^{I\times J}$ is a matrix. TvPDC values form a three-way tensor containing the modes space, time and frequency. The decomposition by means of PARAFAC can conceptually be seen as a multi-linear extension of bilinear decomposition methods such as PCA or ICA. In the PARAFAC model, every entry of a tensor is split into a sum, where each summand is an outer product of loading vectors from each mode. For a third order tensor $X\in R^{I\times J\times K}$ the decomposition of any entry $x_{i,j,k}\in X$ is provided by

$$x_{i,j,k}=\sum_{m=1}^{M}a_{im}\cdot b_{jm}\cdot c_{km}+\eta_{i,j,k},$$

with factor *loadings* or *weights a*_*im*_, *b*_*jm*_, *c*_*km*_ and remaining model residuals *η*_*i*,*j*,*k*_. Similar to the number of components for PCA/ICA, *M* denotes the number of factors within the PARAFAC framework. Model (###4) can equivalently be formulated in tensor form as

$$X=\sum_{m=1}^{M}{\text{A}}_{m}⊗{\text{B}}_{m}⊗{\text{C}}_{m}+H,$$

with loading vectors A_*m*_ = (*a*_1,*m*_,…,*a*_*I*,*m*_)^*T*^, B_*m*_ = (*b*_1,*m*_,…,*b*_*J*,*m*_)^*T*^, C_*m*_ = (*c*_1,*m*_,…,*c*_*K*,*m*_)^*T*^ and tensorial residuum **H $\in R^{I\times J\times K}$** [25]. In general, for an *L*-th order tensor, this model can be extended to

$$X=\sum_{m=1}^{M}{\text{K}}_{m}^{1}⊗\ldots ⊗{\text{K}}_{m}^{L}+H,$$

with X, **H** $\in R^{I_{1}\times I_{2}\times \ldots I_{L}}$ and $K_{m}^{l}\in R^{I_{l}}$.

TvPDC analysis results in a third order tensor $\Pi \in R^{{(D}^{2}-D)\times (N-p)\times F}$ which can be decomposed into products of *M* spatial loading vectors $K_{m}^{S}\in R^{D^{2}-D}$, *M* temporal loading vectors $K_{m}^{N}\in R^{N-p}$ and *M* frequential loading vectors $K_{m}^{F}\in R^{F}$:

$$\Pi =\sum_{m=1}^{M}{\text{K}}_{m}^{S}⊗{\text{K}}_{m}^{N}⊗{\text{K}}_{m}^{F}+H.$$

An essential benefit of model (7) is that instead of comprising (*D* ^2^ − *D*) ∙ (*N* − *p*) ∙ *F* tvPDC values, there are only *M* ∙ ((*D* ^2^ − *D*) + (*N* − *p*) + *F*) remaining factor loadings. This leads to considerably less data than the original raw tvPDC results and is thus substantially easier to handle and interpret.

Furthermore the incorporation of multiple subjects into data analysis is more intuitive than for PCA or ICA, where a model modification for three- (or more) mode data is required. In the PARAFAC framework, the extension from single to group analysis is possible in a straightforward way by including the fourth mode “subject” into the factorization procedure. When data of multiple subjects are available, tvPDC results compose a fourth-order tensor (space, time, frequency and subject). Its decomposition then yields an additional mode comprising a subject-individual weight for every factor.

An unfavorable property of the PARAFAC model is its scale-invariance, as for each factor the multiplication of a loading in one mode can be compensated by analogously dividing the associated loading of any other mode by the same coefficient. For three-way decomposition, this leads to two degrees of freedom. Therefore, we constrained the temporal and frequential loadings to range from zero to one which then allows an intensity interpretation of raw unconstrained spatial loadings.

Two-way decomposition procedures such as PCA and ICA furthermore suffer from rotational invariance of decomposition results, which are overcome by restricting components to be orthogonal or statistically independent. In [26] it is shown that this problem does not occur in PARAFAC decomposition if it holds

$$\sum_{l=1}^{L}\;\text{rank}(K^{l})\geq 2M+(L-1),$$

with factor matrices $K^{l}\in R^{I_{l}\times M}$ containing the factor loading vectors $K_{m}^{l}$. The condition is originally formulated by means of *k-rank* instead of the conventional *rank*. The k-rank is the maximal number *k*_*r*_ such that any subset of *k*_*r*_ columns in the matrix is linearly independent. Thus, inequality (8) is sufficient but not necessary as the rank is never lower than k-rank. Accounting for a suitable interpretability of tvPDC tensor decomposition, the number of factors is normally smaller than *I*_1_,*I*_2_ and *I*_3_ (i.e. number of channel combinations, frequency resolution and number of sample points). Consequently, the rank of all three component matrices is *M* and thus the left side of inequality (8) adds up to 3*M*, whereas the right side equals 2*M* + 2. This indicates that in the context of tvPDC the decomposition is unique up to scaling and permutation if *M* ≥ 2. This constraint also applies if more than three modes are available (e.g. additional mode subject) because the inequality can then be generalized to *M* ≥ (*L* − 1)/(*L* − 2).

The determination of an adequate number of factors *M* is a crucial issue. While a choice which is too few results in an insufficient model fit, too many factors lead to an overestimation where different estimated factors may correspond to the same underlying component or only account for noise. There are several ways to determine the number of factors *M* [27–29]. The appropriateness of the model can for example be evaluated by using heuristics such as Akaike’s information criterion (AIC), Bayesian information criterion (BIC) and Core Consistency Diagnostic, as proposed in [23]; or by generally regarding the sum of squared error, or explained variation, or convergence of the algorithm. Furthermore an observance of the multiple cosine (MC) can help to assess the suitability of the resulting model [30]. As an example, cos(A_*i*_,A_j_) denotes the cosine between *i*-th and *j*-th factor within the first mode of model (5) and provides information on how similar factor *i* and *j* are regarding the first mode. The multiple cosine between *i*-th and *j*-th factor MC_ij_ = cos(A_*i*_,A_j_) ∙ cos(B_*i*_,B) ∙ cos(C_*i*_,C_j_) then provides information on how similar they are regarding all modes. A high absolute value of MC_ij_ indicates that *i*-th and *j*-th factor represent the same underlying component or that obtained solutions are degenerate [30].

The model estimation via minimizing the error term $\eta_{i_{1},i_{2},\ldots ,i_{L}}$ is frequently performed using the alternating least squares (ALS) method. Briefly, this algorithm involves a single least square optimization step for every mode in turn, while the other ones are kept fixed. In this study we used the implementation provided in the *N*-way Toolbox for MATLAB by Andersson and Bro [31]. Due to the positivity of tvPDC values, all weights were constrained to be nonnegative [9, 32].

## Materials

### Simulated Data

**Fig 1:**
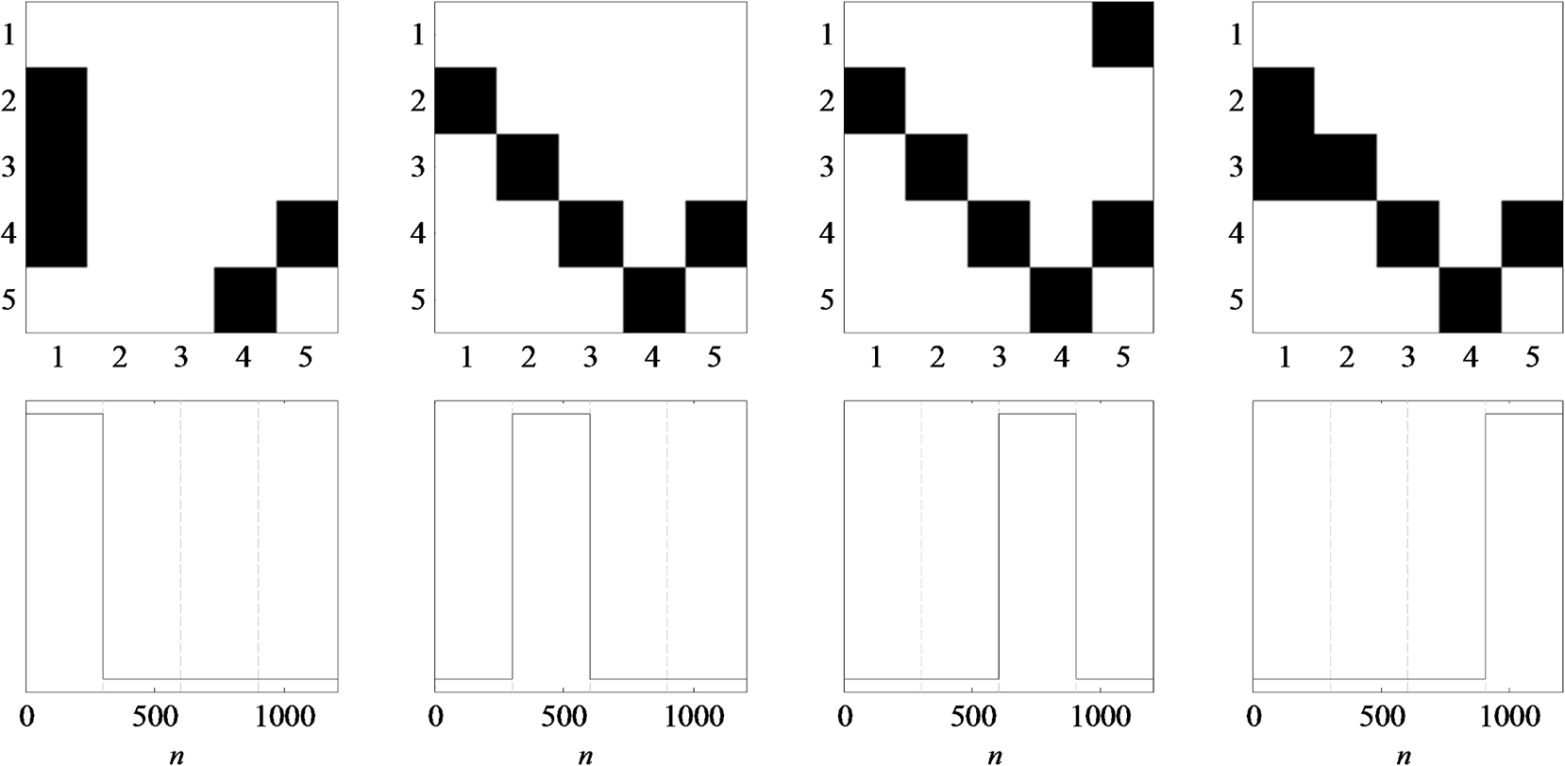
Ground truth model of simulated data. Upper row: spatial loadings; lower row: temporal loadings.

In a first step, we used simulated data with known connectivity structure to reveal the general applicability and effectiveness of PARAFAC analysis in the framework of tvMVAR-based tvPDC analysis. Therefore, we realized a time-variant MVAR process of order *p* = 4 with *D* = 5 nodes, 100 trials and varying number of sample points between *N* = 200 and *N* = 2000 sample points. The underlying ground truth was composed out of four different network constellations, adapted from [2], whereby every constellation was kept fixed for a block of one fourth of the total number of sample points. Adjacency matrices of the model together with the corresponding chronological sequences for *N* = 1200 are illustrated in Fig 1; a black square in the *d*_1_-th row and the *d*_2_-th column of the spatial adjacency matrices indicates a directed interaction from node *d*_2_ to node *d*_1_.

### Real Data

The suitability of tensor decomposition in the case of real data was explored by applying PARAFAC decomposition to an EEG data set of 21 subjects, derived from a study investigating visual evoked potentials. Prior to the experiment detailed information on the aim and the procedures of the experiment was provided to each subject and written informed consent was obtained. The procedure was approved by the Ethics Committee of the Jena University Hospital (reference number 1349-06/04).

Connectivity in the visual system involves ongoing discussion as to whether visual pathways can be divided into two visual subsystems. These are often referred to as the magnocellular system and parvocellular system [33] or the “where” and “what” system [34]; also debated is how connectivity in the visual network is modulated (bottom-up vs. top-down, [35]). The “where” system is thought to assess spatial relationships and object movement, the “what” system is thought to involve the visual identification of colors, patterns or objects. Anatomically, it is assumed that the “what” system involves connections between the occipital and temporal brain areas, whereas the “where” system involves connections between the occipital and parietal brain regions. For this study we thus assume that connections between electrodes P7, P8, O1, Oz, O2 reflect the “what” system, and connections between CP3, CP4, O1, Oz, O2 reflect the “where” system.

Visual evoked potentials were elicited by non-moving sine wave vertical gratings (see Schulte-Körne, Bartling [36] for details regarding stimulus material). Due to the stationarity of visual stimuli, one can expect that connectivity will basically be observed in the “what” system, rather than in the “where” system.

Forty stimuli were presented for 1100 ms each, followed by an interstimulus interval of 900 ms, during which subjects fixated on a cross appearing on a black computer screen. Subjects were instructed to press a button to indicate their perception of the stimulus.

**Fig 2:**
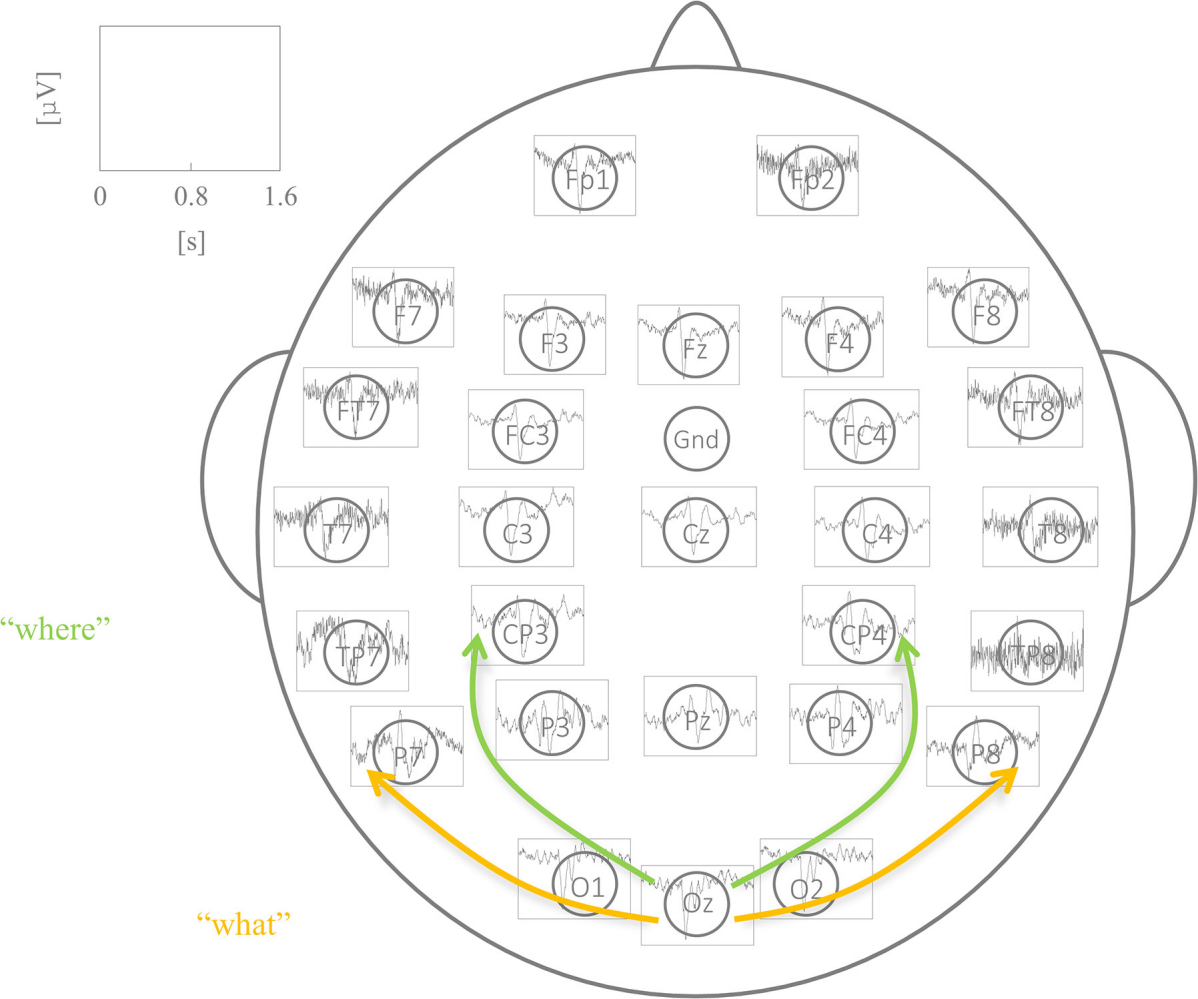
Results of one exemplary subject. EEG data (mean over trials) together with an illustration of the visual “what” and “where” pathways.

EEG data were recorded by Ag/AgCl electrodes over 28 active electrodes, two reference channels, as well as three channels to register eye movement. The real data originate from a study in which three different paradigms were acquired within the same EEG session. These paradigms address different neuronal systems (visual, auditive and linguistic). It was therefore important to cover the respective neuronal networks with the number of electrodes at hand. Thus we chose an enhanced 10/20-system [37], where in addition to the classical 10/20 system, positions FT7, FC3, FC4, FT8, TP7, CP3, CP4, TP8 and Oz were included (see Fig 2). Two electrodes placed at the outer canthi of each eye were used for horizontal eye movement registration. Vertical eye movements were tracked by Fp1 as well as an electrode positioned under the left eye. Electrode impedance was kept beneath 10 kΩ. Data were sampled at a rate of 500 Hz and filtered online with a lowpass of 100 Hz and a highpass 0.1 Hz (Scan, DOSVersion, Compumedics Neuroscan). EEG recordings were referenced to the right mastoid. Left mastoid was also registered for the purpose of re-referencing. Preprocessing was performed offline using VisionAnalyzer. Data were re-referenced against joined mastoids and ocular correction was conducted automatically for horizontal and vertical eye movements using the algorithm of Gratton and Coles [38]. Finally, data were segmented into intervals lasting from 500 ms before and 1100 ms after the stimulus onset and were downsampled to 125 Hz.

The paradigm using stationary sine wave gratings evokes a complex of components comprising positive amplitudes around 150 ms and 250 ms at occipital electrodes (P100 and P200) as well as negative amplitudes around 150 ms at fronto-central electrodes (N100) and 400 ms at centro-parietal electrodes (N400). The applied channel arrangement with the grand average of visual evoked potentials for one subject is depicted on Fig 2. Electrodes which show increased activation are mainly located in occipital, inferior temporal, inferior parietal and frontal brain regions which are associated with the visual network [39].

## Results

### Simulated Data

The selection of the tvMVAR model order *p* must be carefully determined. If *p* is chosen too low, data properties are not considered adequately enough by the model, if too high, too many parameters must then be estimated which can lead to a loss of significance or possible computational problems. However, there is a region between these extremes where different choices of *p* lead to very similar network results [40]. This was confirmed by our fourth-order simulation: for the choice of *p* = 4, all connections were identified correctly; for *p* < 4, connections which rely on an AR parameter of a higher order than *p* were not detected; for *p* > 4 no more connections emerged than for *p* = 4.

The Kalman Filter involves two control parameters *c*_1_,*c*_2_ where *c*_1_ regulates the adaption of the covariance matrix and *c*_2_ defines the step-width of the random walk that is used to update tvMVAR parameters. A suitable indication for an appropriate choice of *c*_1_ and *c*_2_ is provided by minimum mean squared model residuals [40]. Basing on this, the parameters for the simulation data were set to *c*_1_ = *c*_2_ = 0.03.

**Fig 3:**
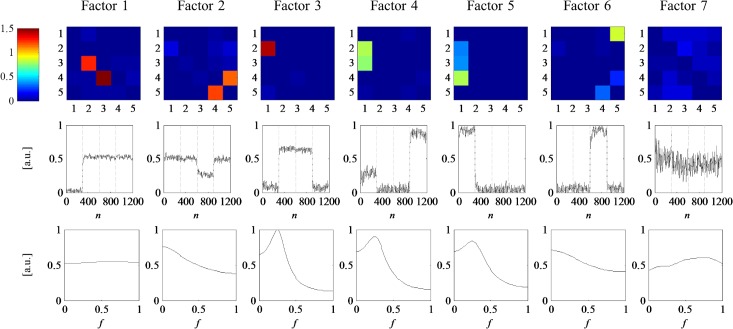
TvPDC tensor decomposition of the simulated data with M = 7 estimated factors. First row: spatial loadings; second row: temporal loadings; third row: frequency loadings.

The number of factors *M* was monitored by means of AIC, BIC, Core Consistency Diagnostic and the sum of squared error. All measures showed a sharp bend around *M* = 6 which suggests that the number of factors should be chosen in this environment. Furthermore, there is a steep rise of the triple cosine in this area, indicating that for higher *M*, different factors become more and more alike. A visual inspection of the results clearly confirms this suggestion: for *M* ≤ 6 all factors correspond to underlying true connectivity patterns, while for any *M* > 6 there are additional components which most likely account for noise. Fig 3 illustrates the results for *M* = 7. Here, the factors are ordered by explained variance, from high to low. The factor with the lowest variance explanation also yields low spatial factor loadings in combination with diffusively distributed temporal and frequential factor loadings. Taken together, this suggests that this factor accounts for background noise. All other factors correctly reflect the true dynamic network, which will be exemplified by means of the second block of the underlying model (Fig 1, second column): interaction 1→2 is spatially reflected by factors 3, 4 and 5. Temporal loadings of block 2 are high for factors 1, 2 and 3. Combining this spatial and temporal information, it becomes apparent that only factor 3 explains the interaction 1→2 in block 2. Analogously, factor 1 accounts for interactions at 2→3 and 3→4, as high spatial loadings at these locations together with high temporal loadings in block two only occur in factor 1. Finally, the remaining two connections 4→5 and 5→4 derive from factor 2 since it is the only factor which combines high weights between nodes 4/5 and high weights within the second block. Following this reasoning, any other of the four ground truth networks can be reconstructed by the first six factors.

The influence of sample size *N* on PARAFAC results accords with the influence of *N* on tvPDC results: an increase of *N* leads to an improved performance. For tvMVAR models, it is well-known that a raised number of samples *N* goes along with an enhanced accuracy of model estimation, leading to a better network identification by means of tvPDC. This is passed on to the benefit of tvPDC-based PARAFAC results, as they are confined by the properness of the precedent tvMVAR model estimation.

The usefulness of tensor decomposition in the context of subsystem analysis was investigated by consecutively analyzing any possible subset of the five nodes. Not surprisingly, a limitation of the five-dimensional simulated data to a subset *before* tvMVAR estimation and tvPDC calculation causes several false-positive tvPDC results due to the hidden sources (if X has an influence on Y and Y has an influence on Z, X seems to influence Z if Y is absent). Thus, subsequent tensor decomposition is rendered superfluous because it would be based on incorrectly detected connectivity patterns. If, however, the limitation to a subset is performed *after* tvMVAR estimation and tvPDC calculation (induced subnetwork), these spurious interactions are circumvented, and the following tensor factorization yields correct results for any choice of subsets.

### Real Data

#### TvPDC results

For empirical data, the correct tvMVAR model order is naturally unknown. Information criteria such as Akaike’s or Bayesian information criterion (AIC, BIC) can roughly indicate a range for an appropriate choice, but should be critically evaluated, as for example by assessing the coincidence between Fourier spectra of the data and estimated AR spectra. For the present EEG data, the suggestions provided by AIC and BIC (AIC = 15, BIC = 8) were too low to allow for a sufficient fit between Fourier spectra of the data and estimated parametric AR spectra. This is why we chose *p* = 20, where similarity between both spectra is satisfactory, while data over-fitting is kept reasonably low.

The acquisition of Kalman control parameters *c*_1_ and *c*_2_ for the empirical data was carried out in the same way as for the simulated data and resulted in *c*_1_ = 0.03 and *c*_2_ = 0.008.

**Fig 4:**
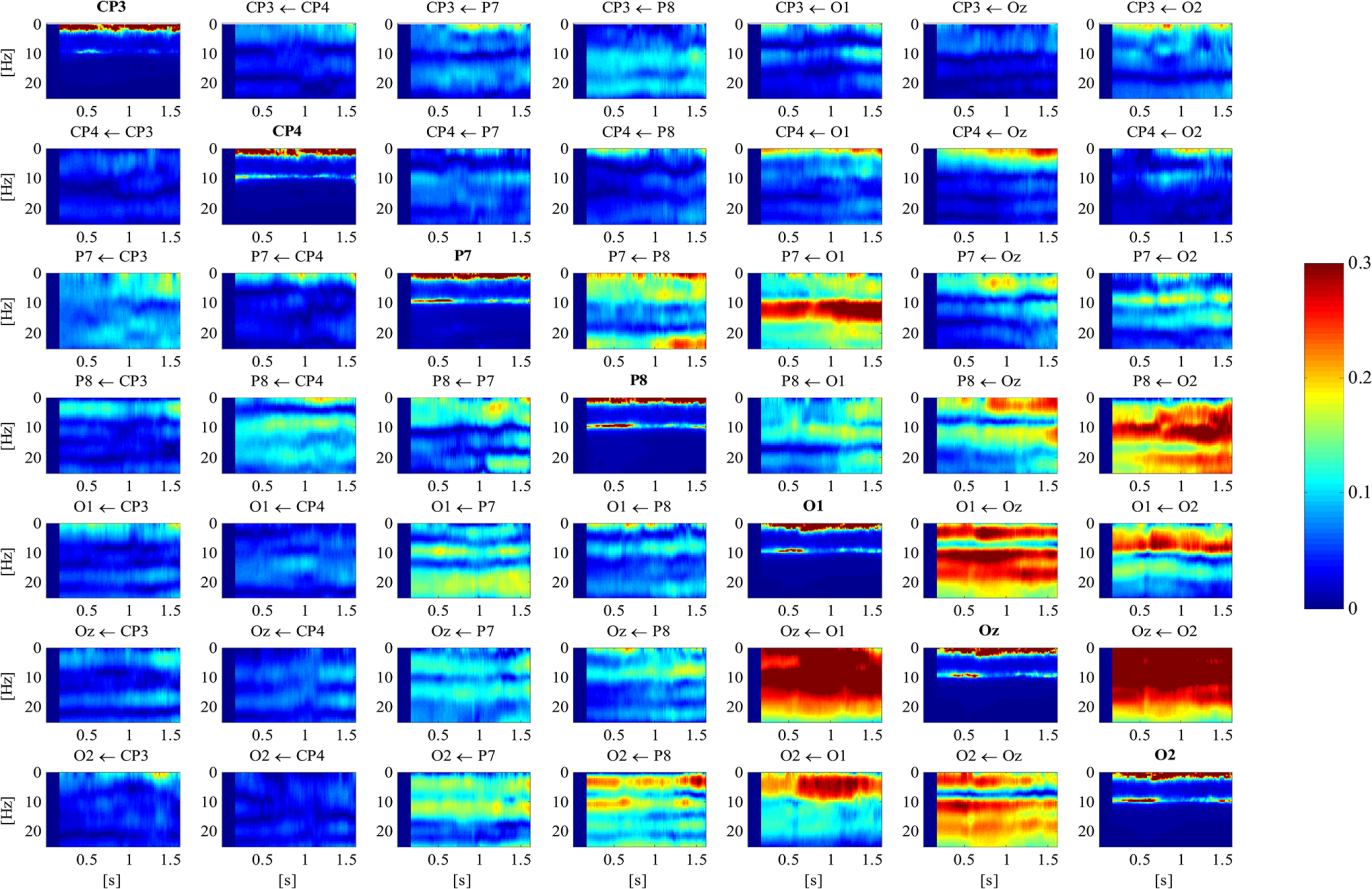
EEG tvPDC results of one exemplary subject between channels CP3, CP4, P7, P8, O1, Oz and O2. Maps on the diagonal depict corresponding parametric AR spectra. Arrows in the headings indicate direction of the interaction. Stimulus onset is at 0.5 s.

The *D* ^2^ − *D* = 20 time-frequency tvPDC matrices of the simulated example can be visually inspected easily, whereas this is not possible anymore for *D* ^2^ − *D* = 756 EEG based tvPDC matrices. However, to provide an impression of the raw results, tvPDC maps of the seven channels representing the previously described “what” and “where” pathways of the visual system, as well as the parametric AR spectra are depicted in Fig 4.

#### Proof-of-principle: decomposition of induced subnetwork

In order to provide a proof-of-principle for the application of PARAFAC to real EEG data, we specifically chose data from an experiment in which the paradigm allows the formulation of strong neurophysiological hypotheses about the expected connections. As described in 3.2, the visual evoked potentials were elicited by non-moving sine wave vertical gratings so that pronounced connectivity was basically expected in the visual “what” system. For this reason, we examined the benefit of restricting tvPDC factorization to an induced network, namely the channels which are involved within the “where” and “what” pathways of visual processing (CP3, CP4, P7, P8, O1, Oz, O2). It is important to emphasize that the tvMVAR model estimation was performed before the subsystem analysis in order to account for the influences of the excluded channels.

The number of factors *M* was determined in consideration of the same quantitative measures as used for the simulated data. We regarded the values of each criterion as a function of chosen factor number *M* where we included *M* = 1,…,20. Basing on this, we considered several aspects. As a first clue (similar to the procedure in cluster analyses) we utilized the discrete second derivative of the resulting functions to attain an impression of an adequate choice of *M*. A maximum value of the second derivate indicates a maximum curvature, i.e. in this context a maximum change of improvement (quantified by the utilized criterion values) when the number of components is increased by one. This led to a choice of *M* = 4. As a second indication, on the other hand, all measures still showed a steep decrease for *M* > 4, pointing towards a higher choice. Third, for *M* > 7 the values of Core Consistency Diagnostic showed an alternating behavior, indicating that results start to be affected by noise. Taking these hints together, our final decision was *M* = 5. This choice was confirmed by visual inspection of the results, as for higher *M*, additional factors did not yield spatial, temporal or frequential patterns which are clearly distinguishable from the first five factors.

**Fig 5:**
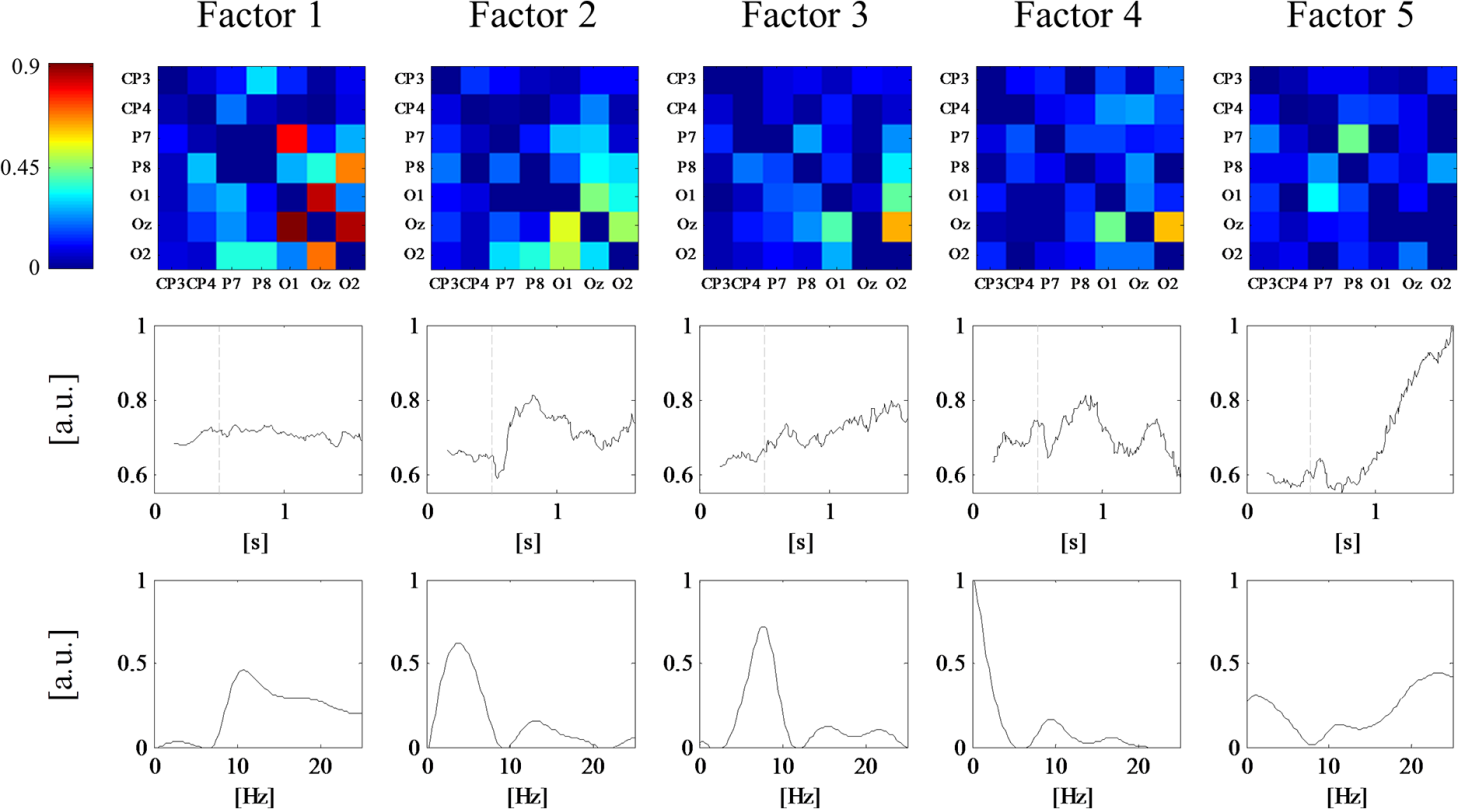
Tensor decomposition of induced tvPDC subset CP3, CP4, P7, P8, O1, Oz, O2 for one exemplary subject. First row: spatial loadings; second row: temporal loadings (dashed line denotes stimulus onset); third row: frequency loadings.

The results of the decomposition of the induced subnetwork are provided in Fig 5. Again, the factors are ordered by variance explanation from high to low. It is apparent that several courses of the temporal mode are related to stimulation onset. The most explicit temporal response occurs in factor two. The corresponding spatial loading map of this factor clearly patterns the occipital and parietal regions which form the “what” pathway, whereas there are no high loadings at the centro-parietal nodes of the “where” pathway. This is in line with the fact that the presented stimuli were non-moving. Frequency curves of this factor show high loadings in the theta band which is commonly associated with working memory [41].

**Fig 6:**
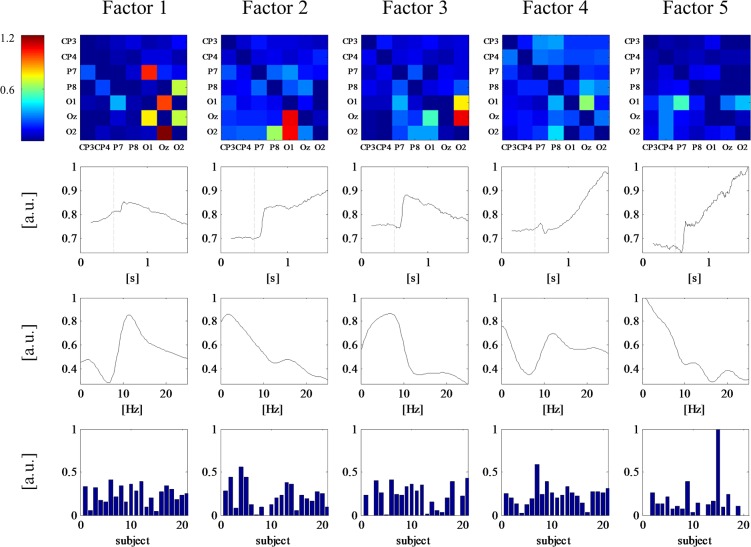
Tensor decomposition of induced tvPDC subset CP3, CP4, P7, P8, O1, Oz, O2 for the whole group. First row: spatial loadings; second row: temporal loadings (dashed line denotes stimulus onset); third row: frequency loadings, fourth row: subject loadings.

The whole group of 21 probands was examined by decomposing the fourth order tvPDC tensor containing the modes space, time, frequency and subject. The results in Fig 6 show that in general, the response to stimulus onset in temporal domain is more pronounced in multi subject decomposition than in single subject decomposition, whereas distinct features in the frequential mode seem to be more smoothed. In all but factor number five, nodes of the “what” network have higher spatial loadings than the “where” nodes. However, it may well be that the last factor is confounded by an artifact because the subject loading of subject number 15 is excessively high. Combining the results of the temporal and spatial loadings, it is thus possible to deduce the occurrence of high connectivities within the "what" system is a response to the stimulus in general, rather than subject-specifically. However, subject loadings indicate slight variability in individual behavior, which suggests that although the *path* of visual information flow applies to any subject, the *degree* of connectivity is subject-specific.

#### Clinical practice: decomposition of the whole network

For the present proof-of-principle study we chose a paradigm which addresses rather basic neurophysiological/visual processes (as described above), thereby we were able to limit the decomposition to a hypothesis-driven subset of tvPDC results containing several electrodes of interest. The reality of most cognitive neuropsychological experiments is however different as oftentimes, it is not possible to formulate such strong hypotheses about the expected network connections. Here, PARFAC offers a solution as an overall view of the results for the complete channel arrangement does not pose a problem. The results of the decomposition for the complete set of our EEG electrodes (provided as supplementary material S1–S3 Figs) can be summarized as follows: on the whole, high spatial weights are mainly located between neighboring electrodes. In particular, there are several factors which additionally comprise pronounced spatial weights within the visual system, complemented by the channels forming the “what” pathway. Yet, there is no factor whose temporal loadings are irrefutably related to the stimulus onset. In other words: while spatial and frequential results of subsystem analysis were confirmed to some extent, the temporal course of the experiment could not be retrieved. On the other hand, when no clear hypothesis on the site of interaction is available, spatial-domain results of the whole decomposition could offer a means for the definition of relevant channels for subsystem analysis.

## Discussion

The application of tvMVAR models with a subsequent calculation of tvPDC is a well-established means to quantify effective connectivity in the neurosciences [1]. Due to its high dimensionality however, visualization and interpretation of results presents a major challenge and extracting the essence of findings is therefore oftentimes hardly feasible. To remedy this problem we proposed an approximation of the resulting tvPDC tensor by a sum of *M* outer products [24, 42]. Thereby, the already introduced decomposition of the time-frequency content in the *signal* [18, 20] is broadened to include time-frequency content in *connectivity* properties as provided by tvPDC.

Within this procedure, resulting tvPDC tensors are rearranged, offering an advanced perspective on the gained connectivity results. This approach is beneficial in various respects. First, real life network constellations are commonly composed of multiple spatial, frequential or temporal components. Oftentimes it is hard to distinguish them by an inspection of tvPDC and in these cases PARAFAC analysis of the results provides a complementary view on the results by the segregation of underlying components into different factors. Second, frequently brain activity is supposed to emerge within spatially limited regions. Sometimes, there is a clear working hypothesis about the channels that are involved in these processes and ought to be included in connectivity analysis. However, in order to avoid spurious interactions due to disregarded nodes, a full multivariate model comprising the whole electrode arrangement is indispensable. A viable option is provided by restricting PARAFAC analysis of multivariate tvPDC results to the decomposition of an induced subset of paradigm-relevant electrodes. The full multivariate model, applied first, incorporates all possible interactions while the subsequent spatially bounded tensor decomposition can reveal subnetwork-immanent connectivity patterns. Third, the rearrangement of tvPDC results drastically reduces the number of output data that have to be examined. This may be the most useful benefit in clinical practice, as oftentimes there is no clear hypothesis about the location of relevant interactions. Then, connectivity maps within the whole set of nodes have to be investigated which is not feasible in most practical applications due to the fairly high number of recorded electrodes. In these cases, the condensation of analysis output by tensor decomposition offers an opportunity to assess an integrative view on the whole network. Furthermore, these decomposition results can subsequently be used to define channels of interest: if one factor shows e.g. a temporal behavior which corresponds to a stimulus, or if the frequential mode of one factor yields high loadings in a particular frequency band, the associated spatial map can be used to select nodes that are fed into an ensuing subsystem decomposition.

Finally, the approach offers a straightforward possibility to extend the analysis of individual subjects to an examination of group data. In contrast to common two-way decompositions, this can be carried out without model modification, since the three-way tvPDC tensor can readily be extended to a four-way tensor with the additional mode “subject”. The decomposition then yields spatial, temporal and frequential weights which apply for the whole group, together with individual subject loadings.

Time-variant five-dimensional simulations were used to demonstrate the general applicability of the proposed procedure. It has been shown that all modeled interaction structures can be re-identified in tvPDC decomposition results if the number of factors *M* is appropriately chosen; we have introduced several quantitative measures which all have proven to provide useful hints for a proper choice of *M*.

EEG recordings during a visual task were used to demonstrate the feasibility and benefit of the approach in the case of empirical data. On the basis of an explicit hypothesis, PARAFAC analysis was restricted to a delimited subset of electrodes *after* full-dimensional tvMVAR estimation and tvPDC calculation. The decomposition outcome clearly revealed several event-related factors, as associated curves in the temporal mode undoubtedly reflected the stimulus onset. Furthermore, the results of the spatial mode supported the assumption that for this experiment, information transfer is mainly located in the “what” pathway rather than in the “where” pathway which is in accordance with the non-moving stimuli of the experimental setup. This finding was substantiated by the extension of PARAFAC analysis to the group of 21 probands, where the stimulus onset in the temporal mode and high loadings of the “what” pathway in the spatial mode were even more distinctive than by the application of subject-individual tensor decomposition.

Oftentimes, it is not possible to establish a hypothesis which enables to focus on several regions of interest. For such cases however, factor decomposition enables a view into the entire channel arrangement. Yet for the present data, it becomes apparent that no clear stimulus-related patterns emerge if the complete set of tvPDC results is decomposed. A reason for this effect may be that the event-induced sub-network is limited only to a small portion of the whole electrode system. Thus, it only accounts for a minor portion of explained variance in model (7) and might therefore be neglected by the ALS estimation. Consequently, quantitatively superior interactions possibly subdue connectivity patterns that are actually relevant for the present topic of interest. However, it also became apparent that even after the decomposition of the whole electrode scheme, high loadings between the nodes comprising the visual areas and the “where” pathway can be retrieved in the spatial mode. In the case when no hypotheses about the location of relevant interactions is available, this in turn enables a data-driven definition of electrodes for a subset analysis, which then allows a more subtle insight into experiment-related connectivity patterns.

Nevertheless, there are several issues which should be kept in mind when applying the above suggested procedure and interpreting the results.

In some cases, there is a considerable difference between EEG amplitudes of several channels and/or several subjects. Scale-invariant alternatives are then required to avoid misinterpretations due to this imbalance in signal power [43]. Therefore, it is necessary to monitor these data properties prior to an interpretation of tvPDC values or any further results basing on them. The individual EEG amplitudes in the present data only showed small variations concerning electrodes and probands. This evaluation was clearly confirmed by a comparison between tvPDC results and the corresponding scale-invariant generalized tvPDC [44], as both measures closely resemble each other for the present data–which consequently also applies to the subsequently performed tensor decompositions.

In this work, quantification of node interaction was executed by means of tvPDC. However, dependent on research questions or data characteristics, tvPDC is not always the most appropriate choice and it is then advisable to prefer other suitable connectivity measures as for instance transfer entropy, Granger causality or directed transfer function [43]. In these cases, too, tensor decomposition could beneficially be applied in order to condense the information contained in network analysis output, as the presented decomposition approach is not limited to tvPDC or tvMVAR in general.

A critical point is that all analyses have been conducted in sensor space. EEG signals are usually affected by volume conduction which induces correlated sensor activities at neighboring sensors by superposition of underlying brain source activities, leading to misinterpretations of obtained connectivity results [45, 46]. It has been demonstrated that procedures like PCA and ICA can beneficially be used to decompose EEG data into source signals and mixing dipole signals [47]. Based on this, connectivity analysis can be performed in source space rather than at sensor level [48]. It is worthwhile to investigate how far a de-correlating preprocessing step or source-modelling affects PARAFAC results. In this context, it is of special interest if the PARAFAC approximation of tvPDC values leads to a model in which connectivity patterns resulting from volume conduction and connectivity patterns based on source activity are separated into different model factors.

Finally, PARAFAC is the last step of a long processing scheme requiring a well-considered choice of an adequate mathematical model, correct model estimation and the selection of an appropriate measure of connectivity. In particular, there are many parameters which have to be tuned during the proposed analysis process. TvMVAR estimation requires appropriate Kalman filter settings that have to balance between fast adaptation and smoothness of estimated AR matrices. Furthermore, a reasonable selection of the model order *p* is mandatory to achieve a sufficient model fit while avoiding an over-estimation [40]. In the same way, the number of factors *M* for PARAFAC estimation must be chosen with care. For both *p* and *M*, there are several concepts to determine the theoretically optimal number [27, 49], but any suggestion should be critically evaluated by the experienced user, as the numerically optimal choice is not always the expediently optimal one.

## Supporting Information

S1 Fig Tensor decomposition of tvPDC for all 28 EEG electrodes and the whole group of subjects. First row: spatial loadings; second row: temporal loadings (dashed line denotes stimulus onset); third row: frequency loadings; fourth row: subject loadings.
(TIF)

S2 Fig Tensor decomposition of tvPDC for all 28 EEG electrodes and the whole group of subjects. MatlabFIG-file of spatial loadings.
(FIG)

S3 Fig EEG channel labels. Ordered electrode designation corresponding to channel combinations within spatial maps of tensor decomposition of tvPDC for all 28 EEG electrodes.
(TIF)
