# Supplementary figures and images for: Advanced Insights into Functional Brain Connectivity by Combining Tensor Decomposition and Partial Directed Coherence
Source: PLoS One. 2015 Jun 5;10(6):e0129293. doi: 10.1371/journal.pone.0129293 (PMC4457931; doi:10.1371/journal.pone.0129293)

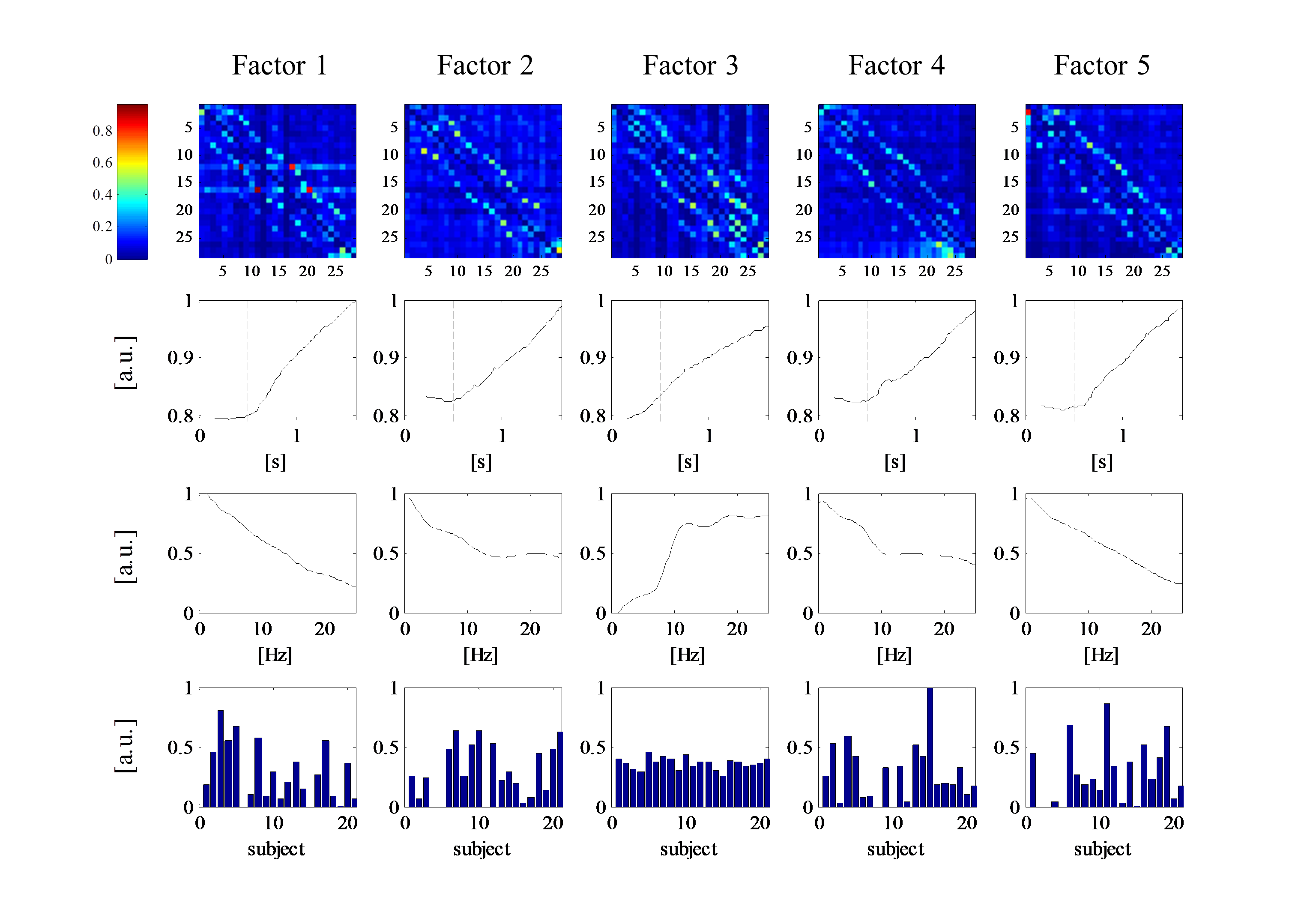

Supplement: S1 Fig — First row: spatial loadings; second row: temporal loadings (dashed line denotes stimulus onset); third row: frequency loadings; fourth row: subject loadings. (TIF) [file pone.0129293.s001.tif]

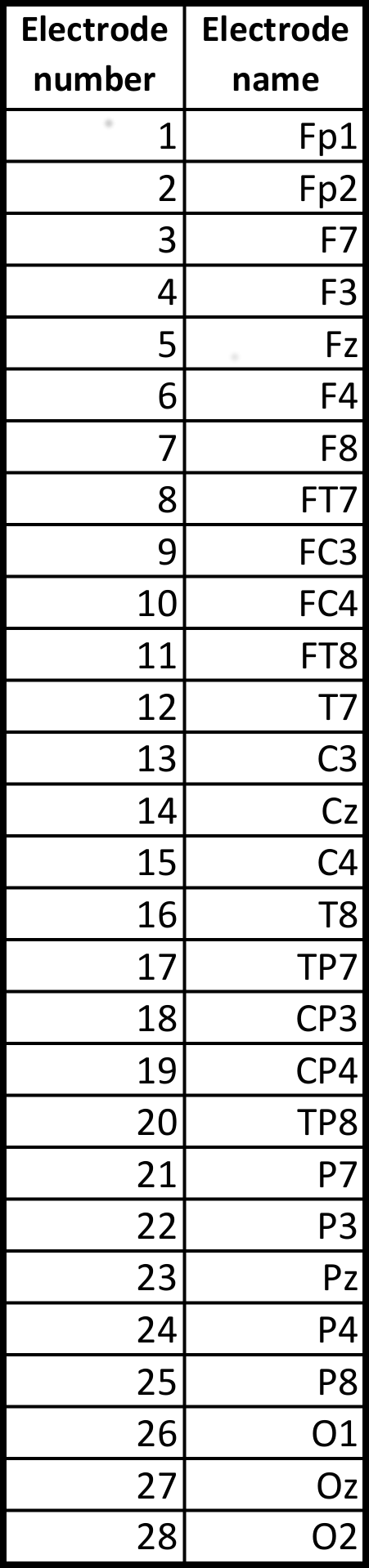

Supplement: S3 Fig — Ordered electrode designation corresponding to channel combinations within spatial maps of tensor decomposition of tvPDC for all 28 EEG electrodes. (TIF) [file pone.0129293.s003.tif]
